# Angiogenic Factors and Renal Disease in Pregnancy

**DOI:** 10.1155/2011/281391

**Published:** 2011-07-28

**Authors:** Julie S. Rhee, Brett C. Young, Sarosh Rana

**Affiliations:** ^1^Department Obstetrics and Gynecology, Beth Israel Deaconess Medical Center, Harvard Medical School, Boston, MA 02215, USA; ^2^Division of Maternal Fetal Medicine, Department Obstetrics and Gynecology, Beth Israel Deaconess Medical Center, Harvard Medical School, Boston, MA 02215, USA

## Abstract

*Background*. Preeclampsia is difficult to diagnose in patients with underlying renal disease and proteinuria. Prior studies show that there is an angiogenic factor imbalance with elevated levels of antiangiogenic proteins soluble fms-like tyrosine kinase 1 (sFlt1) and soluble endoglin (sEng) and reduced levels of the proangiogenic protein, placental growth factor (PlGF) in women with preeclampsia. These angiogenic biomarkers may be useful in distinguishing preeclampsia from other conditions of pregnancy, which may present with overlapping clinical characteristics. *Cases*. Case 1: A multiparous woman at 18 weeks gestation with nephrotic syndrome presented with hypertensive emergency and worsening renal insufficiency. She underwent induction of labor for severe preeclampsia. Her sFlt1 and sEng levels were at the 97 percentile while her PlGF level was undetectable (less than the 1st percentile). Case 2: A nulliparous woman with lupus nephritis at 22 weeks gestation presented with fetal demise and heart failure. Three weeks previously, the patient had developed thrombocytopenia and hypertensive urgency. She underwent dilation and evacuation. Her angiogenic profile was consistent with severe preeclampsia. *Conclusion*. Angiogenic factors may provide evidence to support a diagnosis of preeclampsia in patients with preexisting renal disease and proteinuria, conditions in which the classical definition of hypertension and proteinuria cannot be used.

## 1. Introduction

Preeclampsia affects 5–7% of all pregnancies and is a leading cause of maternal and neonatal morbidity and mortality. The classical diagnosis of preeclampsia is made when pregnant women present with new-onset hypertension (140 mm Hg systolic or higher or 90 mm Hg diastolic or higher) and proteinuria (0.3 grams protein or higher in a 24-hour urine specimen) typically after 20 weeks of gestation [1]. However, preeclampsia can be challenging to diagnose using these clinical criteria when a patient has preexisting renal disease which itself causes hypertension or proteinuria. 

The exact pathophysiology of preeclampsia remains unclear and is a leading area of investigation. Recent studies have demonstrated angiogenic imbalance in patients with preeclampsia. Weeks before the onset of preeclampsia, circulating levels of the antiangiogenic factors soluble fms-like tyrosine kinase 1 (sFlt1) and soluble endoglin (sEng) rise to abnormally high levels while levels of the proangiogenic placental growth factor (PlGF) are reduced [2]. 

In this paper, we present two patients with underlying renal disease and proteinuria who were diagnosed with severe preeclampsia. Because of their preexisting medical conditions and the fact that clinical onset of disease occurred in the late second trimester, the diagnosis of preeclampsia was challenging. IRB/Ethics Committee decided approval was not required for this study.

**Case 1**

A 34-year-old gravida 2 para 1 at 17 weeks 6 days of gestation presented to the emergency room from an office evaluation with hypertensive urgency. Her blood pressure on arrival was 164/110 mm Hg. The patient had known nephrotic syndrome with a baseline creatinine of 1.4 mg/dL and a 24-hour urine with 3.7 grams of protein prior to pregnancy. She had no preexisting hypertension. A week prior to presentation, her creatinine had risen to 2.5 mg/dL and her 24-hour urine revealed 9.1 grams of protein. The patient's elevated blood pressures were treated with labetalol. Her worsening hypertension and renal function at the time had been attributed to worsening renal disease due to pregnancy. On presentation to the emergency room, she complained of a mild headache but denied any visual change or epigastric pain. Her physical exam was significant for 3+ deep tendon reflexes and 3+ pitting edema bilaterally.

The patient's obstetric history included a preterm cesarean delivery at 24 weeks for severe preeclampsia with HELLP syndrome. Given her current presentation and her obstetric history, the patient was admitted and placed on intravenous magnesium sulfate for seizure prophylaxis. She had a strong desire to continue her pregnancy. During the hospital course, her blood pressures required multiple intravenous antihypertensive medications. She developed elevated transaminases, thrombocytopenia, and worsening renal function. Serum creatinine was elevated at 2.6 mg/dL, and uric acid was 7.0 mg/dL.

The patient was counseled regarding the poor prognosis of the pregnancy at this previable gestational age and the maternal risks associated with expectant management of severe preeclampsia. The patient chose to undergo an induction of labor at 18 weeks gestation.

Postpartum, her blood pressures, and elevated transaminases normalized, and her creatinine returned to baseline. Placental pathology showed a preterm placenta with accelerated villous maturation, increased perivillous and intravillous fibrin deposition, multifocal extravillous cytotrophoblast proliferation, and erythroblastosis.

**Case 2**

A 35-year-old gravida 1 para 0 with lupus nephritis presented to the obstetrical triage unit at 21 6/7 weeks of gestation with shortness of breath. Her medical history was significant for systemic lupus erythematosus (SLE) with renal and hematological manifestations.

The patient had been recently admitted to the antepartum service at 19 weeks 4 days of gestation for worsening hypertension and thrombocytopenia. During her admission, her blood pressure reached a maximum of 170/110 mm Hg and her platelets reached a nadir of 50 K/uL from 150 K/uL baseline prior to pregnancy. A 24-hour urine was significant for 3.4 grams of protein, increased from a baseline of 1.6 grams prior to pregnancy. She was started on two oral antihypertensive medications and was administered oral and intravenous steroids for what was thought to be an acute lupus flare. The worsening proteinuria, thrombocytopenia, and hypertension were thought to be secondary to worsening SLE. 

Following her first antepartum discharge, the patient presented to triage with new-onset shortness of breath and decreased breath sounds bilaterally on physical examination. A chest X-ray showed cardiomegaly and evidence of congestive heart failure. Transabdominal ultrasound revealed an intrauterine fetal demise. The patient underwent an uncomplicated dilation and evacuation. Further workup of acute heart failure revealed an ejection fraction of 30–35% as well as pulmonary edema. She was admitted to the intensive care unit for close supportive management. The patient's blood pressure improved requiring fewer medications for control. Placental pathology showed extensive involvement of perivillous fibrin, hematomas, and infarcts. The fetus and placenta were growth restricted.

Both patient's clinical presentations presented diagnostic challenges for experienced clinicians. We hypothesized that these patients will have an angiogenic profile unlike a normal pregnancy, but similar to patients with severe preeclampsia. We collected serum samples from both patients at the time of clinical presentation; enzyme-linked immunosorbent assays (ELISA) for sFlt1, sEng, and PlGF were performed with commercially available kits (R&D systems Inc, Minneapolis, MN). All assays were performed in duplicate and values averaged. The correlation coefficient between duplicate results for all three biomarkers was 0.99. These levels were compared to those of gestational age-matched women from a random sample of 2200 women in the trial of Calcium for Preeclampsia Prevention cohort who were normotensive at the time of the blood collection [3]. 

We found that the levels of sFlt1 and sEng were at or above the 95th percentile based on gestational age. Additionally, PlGF levels were below the detection limit (15.6 pg/mL) in both patients, which corresponded to less than the 1st percentile as matched for gestational age (Table 1). We collected this information in the hope that angiogenic profiles may be used to assist the clinicians to confirm a challenging clinical diagnosis of severe preeclampsia even at an early gestational age and aid in timely intervention in these patients.

## 2. Comment

The classic diagnosis of preeclampsia is based on new-onset proteinuria and hypertension in the pregnant patient after 20 weeks of gestation [1]. Preeclampsia prior to 20 weeks of gestation is rare but can occur in severe cases. Patients with underlying renal disease and proteinuria prior to pregnancy make the diagnosis of preeclampsia especially challenging for the clinician. It is important to correctly distinguish between proteinuria and hypertension secondary to chronic renal disease from proteinuria and hypertension as a result of preeclampsia because appropriate treatment may be either delivery in the setting of preeclampsia or supportive management in the setting of chronic renal disease. Women with preexisting renal disease are at increased risk for developing preeclampsia, but not all women with renal disease develop this complication. 

Both patients in our report had underlying renal disease. The first patient had nephrotic syndrome. Although the patient's new-onset hypertension and her past history of a preterm delivery for preeclampsia supported preeclampsia as the diagnosis, her underlying renal disease as well as the early gestational age at presentation made the clinical diagnosis challenging. 

The second patient had a longstanding history of SLE with lupus nephritis and thrombocytopenia as manifestations of her lupus. She had baseline proteinuria of 1.6 grams over 24 hours prior to pregnancy. Pregnancies complicated by lupus nephritis are at increased risk of developing preeclampsia; similarly, there is a significant risk of lupus flare during pregnancy [4]. This patient's presentation made it difficult to distinguish a lupus flare from preeclampsia at 20 weeks gestation; both differential diagnoses were likely. Although this patient's risk of developing preeclampsia was high, her clinical presentation was initially attributed to a lupus flare. In both patients, the angiogenic profile supported the diagnosis of preeclampsia with markedly elevated levels of sFlt1 and sEng and undetectable PlGF levels. 

Angiogenic biomarkers have demonstrated utility in distinguishing preeclampsia from large cohorts of normotensive patients [2]. In prospective cohort studies, the ratio of circulating anti- and proangiogenic protein levels identifies women with early onset preeclampsia with very high sensitivity and specificity [5, 6]. Additionally, further reports have used angiogenic biomarkers to help diagnose conditions with sign and symptoms similar to preeclampsia that limit the usefulness of the classical definition such as thrombocytopenia [7], lupus [4], and renal diseases [8]. 

Qazi et al. demonstrated a difference in angiogenic factors among 52 patients with lupus who developed preeclampsia versus those that did not develop preeclampsia; the authors concluded that angiogenic biomarkers could be used prospectively to distinguish women with lupus at high risk for developing preeclampsia. Women with lupus who developed preeclampsia had significantly higher levels of sFlt1 versus patients with lupus but without preeclampsia [4]. The sFlt1 levels in our patient with lupus were higher than the cohort of patients in Qazi's study.

Additionally, Williams et al. reported a case of a 29-year-old woman with lupus and renal failure at 20 weeks 6 days gestation who presented with renal failure. The patient's diagnosis of preeclampsia was difficult to confirm clinically, given her preexisting renal disease. The patient's sFlt1 levels were high, and PlGF levels in the serum were low, characteristic of the angiogenic profile of preeclampsia [9]. Similarly, Shan et al. reported a case of a 34-year-old pregnant woman at 29 weeks gestation with chronic hypertension and end-stage renal disease on dialysis. She underwent an emergent cesarean section for worsening hypertension. Soluble Flt1 and soluble endoglin levels were normal for gestational age, however, suggesting that the hypertension noted during pregnancy may not have resulted from preeclampsia [8]. Recently, Young et al. demonstrated that angiogenic biomarkers may be used to distinguish gestational thrombocytopenia from thrombocytopenia caused by HELLP syndrome [7]. 

The clinical diagnosis of preeclampsia is challenging to confirm or exclude in patients with underlying renal disease and proteinuria who also develop hypertension. However, accurate diagnosis is critical for appropriate obstetrical management particularly at early gestational ages. 

Our two cases demonstrate that the measurement of angiogenic biomarkers may help clinicians diagnose preeclampsia in patients with underlying renal disease with proteinuria and hypertension that overlap with the classic diagnosis of preeclampsia. This is especially useful at previable gestational ages where accurate and timely diagnosis is critical, as there is no fetal benefit from prolongation of pregnancy. Prospective studies are needed to better characterize the utility of these angiogenic biomarkers in real-time clinical situations in which the patient's comorbidities make the classic diagnosis of preeclampsia difficult to confirm.

## Figures and Tables

**Table 1:** 

	Gestational age (weeks)	Patient's sFlt1 level (ng/mL)	95–97th percentile for sFlt1 in normal pregnancies (ng/mL)*	Patient's sEng level (ng/mL)	95–97th percentile for sEng in normal pregnancies (ng/mL)*	Patient's PlGF level (pg/mL)	5th–1st percentile for PlGF in normal pregnancies (pg/mL)*
Case 1	17.6	8.7	7.5–11.1	16.5	6.9–9.3	5	77–53
Case 2	21.6	21.7	7.7–9.1	120.1	6.6–10.1	3	136–103

*The percentiles for normal pregnancies were obtained from the CPEP study [3]. The values at 18 weeks are given for comparison with patient values at 17.6 weeks; values at 21-22 weeks are given for comparison with patient values at 21.6 weeks. PlGF: placental growth factor; sEng: soluble endoglin; sFlt1: soluble fms-like tyrosine kinase-1.
